# Recruitment of Mediator Complex by Cell Type and Stage-Specific Factors Required for Tissue-Specific TAF Dependent Gene Activation in an Adult Stem Cell Lineage

**DOI:** 10.1371/journal.pgen.1005701

**Published:** 2015-12-01

**Authors:** Chenggang Lu, Margaret T. Fuller

**Affiliations:** Departments of Developmental Biology and of Genetics, Stanford University School of Medicine, Stanford, California, United States of America; Cardiff University, UNITED KINGDOM

## Abstract

Onset of terminal differentiation in adult stem cell lineages is commonly marked by robust activation of new transcriptional programs required to make the appropriate differentiated cell type(s). In the *Drosophila* male germ line stem cell lineage, the switch from proliferating spermatogonia to spermatocyte is accompanied by one of the most dramatic transcriptional changes in the fly, as over 1000 new transcripts turn on in preparation for meiosis and spermatid differentiation. Here we show that function of the coactivator complex Mediator is required for activation of hundreds of new transcripts in the spermatocyte program. Mediator appears to act in a sequential hierarchy, with the testis activating Complex (tMAC), a cell type specific form of the Mip/dREAM general repressor, required to recruit Mediator subunits to the chromatin, and Mediator function required to recruit the testis TAFs (tTAFs), spermatocyte specific homologs of subunits of TFIID. Mediator, tMAC and the tTAFs co-regulate expression of a major set of spermatid differentiation genes. The Mediator subunit Med22 binds the tMAC component Topi when the two are coexpressed in S2 cells, suggesting direct recruitment. Loss of Med22 function in spermatocytes causes meiosis I maturation arrest male infertility, similar to loss of function of the tMAC subunits or the tTAFs. Our results illuminate how cell type specific versions of the Mip/dREAM complex and the general transcription machinery cooperate to drive selective gene activation during differentiation in stem cell lineages.

## Author Summary

Selective gene expression is crucial to making different cell types over the course of the development of an organism. In stem cell lineages, precursor cells terminally differentiate into defined cell types, with onset of terminal differentiation associated with activation of stage- and cell type-specific transcriptional programs. When spermatogonia initiate differentiation and become spermatocytes in the *Drosophila* male germ line, they undergo the most dramatic transcriptional changes that occur in the fly, as over 1000 new transcripts turn on in preparation for meiosis and the striking morphological changes that produce sperm. This robust spermatocyte transcription program requires cooperative action of a testis-specific protein complex, tMAC and the testis-specific basal transcription machinery TFIID. Here we show that the transcriptional co-activator complex, Mediator is key in connecting the two classes of players. We found that Mediator is recruited to spermatocyte chromatin through the interaction of its subunit, Med22 and a putative transcription activator in tMAC. Recruitment of Mediator is then required for proper localization and function of the testis-specific TFIID complex to initiate gene transcription for spermatid differentiation, illuminating how transcription factors and cell type-specific versions of the general transcription machinery cooperate to drive gene activation during differentiation in adult stem cell lineages.

## Introduction

Developmental control of cell type specific gene expression programs is crucial to differentiation in embryonic and adult stem cell lineages. Developmental signaling pathways are ultimately interpreted in the context of cell type-specific chromatin states and by transcription machinery to establish the intricate patterns of gene expression unique to each differentiating cell type [1,2]. Emerging evidence suggests that Mediator, a large, multiprotein complex that integrates transcriptional enhancing and repressing signals from transcription factors, chromatin modifiers, non-coding RNAs and elongation factors to deliver a calibrated output to the transcription machinery to modulate gene expression [3,4], plays critical roles in tissue and cell type specific gene expression programs in metazoans. For example, Mediator-enriched super enhancers contribute to regulation of key cell identity genes in ES cells and many differentiated cell types [5]. Although Mediator was reported to be essential for ESC maintenance and embryonic development [6,7,8,9], and widely involved in human diseases and different types of cancer [3,10], the role(s) of Mediator in adult stem cell lineages are not well understood.

We investigated the function of Mediator in activating expression of a cell type specific transcription program for terminal differentiation in a model adult stem cell lineage, spermatogenesis in *Drosophila*. To initiate differentiation in this lineage, germ line stem cells divide asymmetrically, each producing one daughter that self-renews and one daughter that initiates a series of four spermatogonial mitotic transit amplifying divisions. The resulting 16 interconnected spermatogonia then undergo premeiotic S phase and become spermatocytes [11]. One of the most dramatic cell type specific gene expression programs of the fly initiates at the spermatocyte stage, during which over 2000 genes are transcriptionally activated in meiotic prophase, many for the first time in development [11,12].

Mutations in several genes cause failure to activate many genes in this transcription program and a meiotic arrest phenotype: mutant testes filled with mature primary spermatocytes that fail to enter the meiotic divisions or initiate spermatid differentiation [13]. Molecular cloning and analysis revealed that proper activation of transcription of these terminal differentiation genes in spermatocytes depends on cooperative action of two classes of meiotic arrest genes, expressed specifically in spermatocytes, which encode homologs of either TBP-associated factors (tTAFs) [14,15] or components of the testis meiotic arrest complex (tMAC), a testis-specific version of the mammalian MIP/dREAM and the *C*. *elegans* SynMuvB complexes [16,17,18,19,20,21,22,23,24]. tMAC contains at least 3 potential DNA binding components, Comr, Topi and Tomb [16,18,19,20], as well as several subunits implicated in chromatin remodeling or performing structural roles within the complex [16,22]. It has been suggested that the tMAC component *aly* may help remodel spermatocyte chromatin for global activation of the spermatocyte transcription program [22]. Action of the tMAC complex is needed for transcription of the G2 cell cycle regulators Cyclin B, *cdc25/*twine and *boule* in spermatocytes and of a large set of spermatid differentiation genes [16,17,18,19,20,21,22,23,24], the tTAFs are required for full activation of the spermatid differentiation genes but are dispensable for expression of transcripts for the G2 cell cycle regulators [14,23].

Expression of both the tTAFs and testis-specific components of the tMAC complex is turned on in early spermatocytes but the two classes of genes do not depend on each other to be transcribed [14,15,22,25]. Recruitment of the tTAF protein Sa to promoters of target spermatid differentiation genes required function of the tMAC component, Aly [26]. Several additional meiotic arrest genes not directly involved in regulation of the spermatocyte transcription program have also been discovered [27,28,29].

Here we show that Mediator plays a key role in activating expression in spermatocytes of a large number of transcripts involved in meiotic cell cycle progression and spermatid differentiation. We found that, for several of the many Mediator subunits, spermatocyte specific RNAi knock down produced a range of meiosis I maturation arrest phenotypes in male germ cells. Knockdown of the mediator subunit Med22 by RNAi in spermatocytes resulted in a consistent meiotic arrest phenotype similar to the tTAF mutants, suggesting that Mediator may function with the tTAFs and tMAC to activate the transcription program for terminal differentiation. Expression of *Drosophila* Mediator complex components becomes upregulated in early spermatocytes just prior to expression of the tTAFs, and Mediator subunits colocalized with tTAFs in spermatocytes. Localization of Mediator subunits to chromatin in spermatocytes depended on tMAC but not tTAF function, while spermatocyte specific knockdown of Med22 by RNAi abolished localization of tTAFs to chromatin, suggesting that Med22 may recruit or stabilize tTAFs to chromatin for activation of transcription of differentiation genes. Consistent with this pathway, expression of most spermatid differentiation transcripts dependent on tMAC and the tTAFs also required function of Med22 in spermatocytes. Strikingly, expression of transcripts up regulated in early spermatocytes that did not depend on tMAC and the tTAFs remained largely unaffected in Med22 knockdown testes, suggesting that Mediator is not required for all activated transcription in spermatocytes, but mainly for turning on the developmentally controlled transcription program that depends on tMAC and tTAF. The Zn finger protein Topi, a component of tMAC, interacts structurally with Med22 and may recruit Mediator to target genes. Our results suggest that Mediator serves as a key component in a gene regulatory cascade of transcription activation that establishes the expression program for terminal differentiation in the male germ line adult stem cell lineage.

## Results

### Loss of Med22 function in spermatocytes results in meiotic arrest

**Fig 1:**
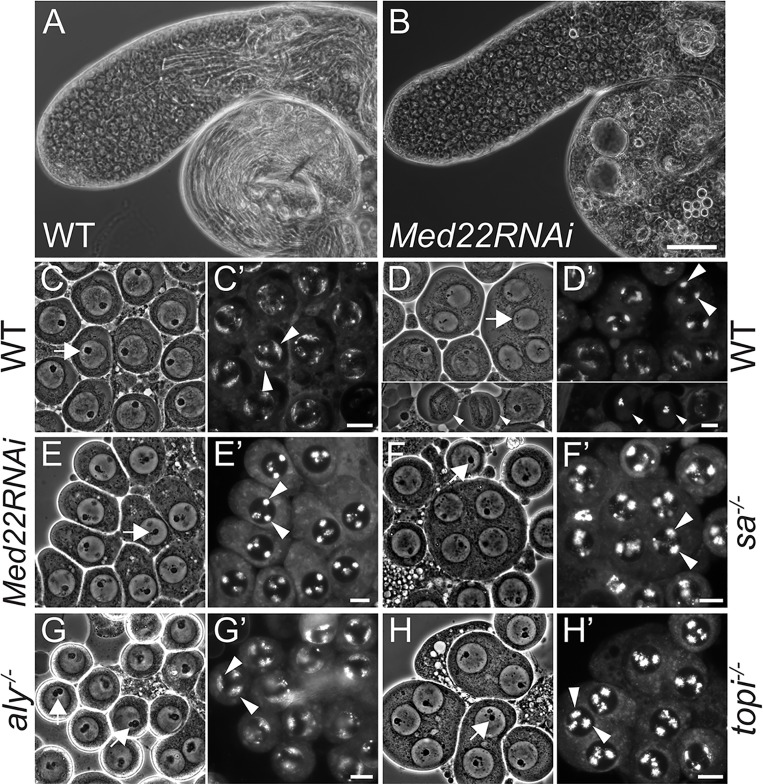
Meiotic arrest due to loss of Med22 function in spermatocytes. **(A, B)** Phase contrast images of testes from (A) wild type and (B) *Med22RNAi* (Bam-Gal4;UAS-Med22RNAi) flies. Bar: 100 μm. **(C-H’)** representative (C-H) phase contrast and (C’-H’) Hoechst stain images of (C-C’) wild type mature spermatocytes, (D-D’) upper panels: wild type spermatocytes entering meiosis I, lower panels: wild type spermatocytes at metaphase of meiosis I or of the arrested spermatocytes of (E-E’) *Med22RNAi*, (F-F’) *sa* ^*-/-*^, (G-G’) *aly* ^*-/-*^ and (H-H’) *topi* ^*-/-*^ mutants. Arrows, nucleolus; Large arrowheads, condensed autosomal bivalents; Small arrowheads, dividing primary spermatocytes at metaphase of meiosis I. Bar: 10 μm.

Function of the Mediator complex in differentiating male germ cells was demonstrated by targeted knock down of individual Mediator subunits specifically in spermatocytes by driving RNAi hairpin expression in late spermatogonia and spermatocytes under control of the germ cell specific driver Bam-Gal4 [30]. Individual knockdown in spermatocytes of several Mediator subunits by RNAi resulted in meiotic arrest of various severity and penetrance (S1 Table). Among the Mediator subunit knockdowns that showed meiotic arrest, *Med22RNAi* resulted in the strongest phenotype at high penetrance: Spermatocytes failed to enter the meiotic divisions and cells arrested at the G2/M transition of meiosis I, accumulating to fill the testis before eventually degenerating near the basal end of the testis (Fig 1A and 1B). Knock down other Mediator subunits, including Med17, Med11 and Med27 also showed meiotic arrest, although the phenotype was less penetrant (S1 Table). Consistent with meiotic arrest, western blots to detect Boule protein, a key regulator of the meiotic cell cycle [31], revealed that testes in which Med22 had been knocked down in spermatocytes completely lacked Boule expression (S1A Fig). Boule protein was reduced but not absent in testes in which Med17, Med11 or Med27 had been knocked down (S1A and S1B Fig), consistent with the lower penetrance.

Analysis of unfixed squashed testes samples stained with Hoechst to visualize chromosomes revealed that spermatocytes in which Med22 had been knocked down by RNAi arrested with partially condensed bivalents resembling the arrest observed in spermatocytes lacking function of the testis TAF gene *sa*. In wild type mature spermatocytes entering the G2/M transition of meiosis I, autosomal bivalent chromosomes, visualized by Hoechst stain of unfixed squashed preparations, initially appear as crescent shaped structures near the nuclear periphery (Fig 1C’, arrow heads) then rounded up and become highly condensed at the nuclear periphery (Fig 1D’, large arrow heads) as the nucleolus starts to break down (Fig 1D, arrow). The wild type condensed bivalents then quickly move toward the middle of nucleus, a transient process rarely captured in wild type testis squashes, while the meiotic spindle sets up for metaphase of meiosis I (Fig 1D and 1D’, lower panels: small arrow heads).

In the arrested *Med22RNAi* spermatocytes, bivalent chromosomes partially condensed, rounded up, and moved slightly away from the periphery of the nucleus (Fig 1E’, arrow heads) while the nucleolus failed to break down (Fig 1E, arrow), similar to mature spermatocytes in flies mutant for *sa* (Fig 1F and 1F’). The meiotic arrest phenotype resulting from null mutants of *topi*, which encodes a Zinc-finger subunit of tMAC, was very similar to that of *Med22RNAi* or of tTAF mutants, with bivalents condensed to spherical shapes slightly away from nuclear periphery and failure of the nucleolus to fully break down (Fig 1H and 1H’ and [18]). Spermatocytes from flies mutant for the core tMAC subunit Aly also arrested at the G2/M transition of meiosis I, but with clear differences in chromatin appearance. In addition, in arrested *aly* mutant spermatocytes, the nucleolus was not circular in shape but had lobe-like extensions (arrows in Fig 1G and [26]), and the condensing autosomal bivalents largely remained as incompletely condensed crescents near the nuclear periphery (arrow heads in Fig 1G’ and [22]), suggesting the *aly* mutant germ cells may either arrest at an earlier time point or with a slightly different, perhaps broader, spectrum of defects than spermatocytes lacking function of tTAFs, *topi* or Med22.

### Mediator proteins localize to condensing chromatin in spermatocytes

**Fig 2:**
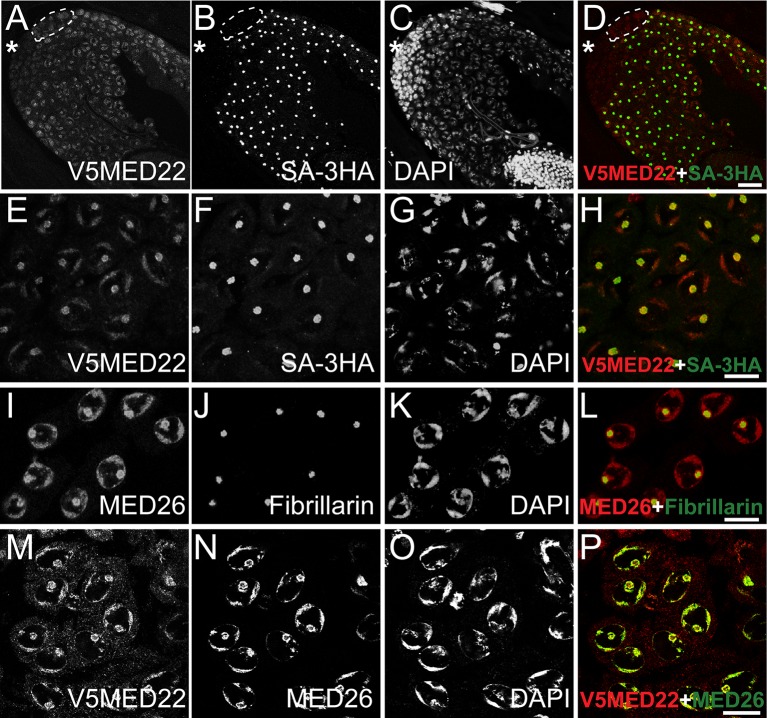
Mediator proteins colocalize with the tTAF protein, Sa to chromatin and the nucleolus in spermatocytes. **(A-H)** Indirect immunofluorescence of (A-D) apical part of testis and (E-H) spermatocytes stained for (A and E) anti-V5 to detect V5-MED22, (B-F) anti-HA to detect SA-3HA, (C-G) DAPI and (E and H) merge, red: V5-MED22, green: SA-3HA. Asterisks: tip of testis. Dashed circle: cyst of spermatocytes expressing MED22 but not SA-3HA. **(I-L)** Indirect immunofluorescence of wild type spermatocytes stained for (I) anti-MED26, (J) Fibrillarin, (K) DAPI and (L) merge, red: anti-MED26, green: Fibrillarin. **(M-P)** Indirect immunofluorescence of spermatocytes stained for (M) anti-V5 to detect V5-MED22, (N) anti-MED26, (O) DAPI and (P) merge, red: V5-MED22, green: anti-MED26. Bars in D: 25 μm, in H, L, P: 10 μm.

Expression of Med22 transcript and protein was lower in stem cells and spermatogonia near the testis apical tip and increased as germ cells became spermatocytes (Figs 2A–2D and S2A). When expression of Med22 protein was visualized by immunostaining with anti-V5 in testes from flies carrying a transgene encoding a V5-MED22 rescuing fusion protein expressed under control of the Med22 promoter plus regulatory elements from the Med22 genomic region (see Materials and Methods), V5-MED22 protein levels were upregulated immediately before Sa protein expression became visible. For example, the cyst marked with a dashed outline in Fig 2A showed upregulation of V5-MED22 prior to appearance of SA-3HA, expressed from a genomic transgene with all the necessary sequences to rescue *sa* null mutants [15]. In spermatocytes, V5-MED22 localized to the condensing chromatin and to the nucleolus, a pattern reminiscent of the localization pattern of tTAF proteins [15,32]. Indeed, V5-MED22 colocalized with the tTAF protein Sa, visualized by anti-HA immunostaining for SA-3HA, in spermatocytes (Fig 2E–2H).

Immunostaining with antibodies against either the endogenously encoded proteins or epitope tagged proteins expressed from genomic transgenes revealed that the MED26 and MED27 Mediator subunits were also expressed in all germline and somatic cells in the apical testis, and that both Med26 and Med27 proteins were upregulated in early spermatocytes, similar to MED22 (S3A–S3F Fig). Immunostaining with anti-MED26 revealed that the protein gradually localized and enriched to the condensing chromatin and the nucleolus as young spermatocytes develop and mature (Figs 2I–2L and S3G–S3K). Co-immunostaining with anti-V5 and anti-MED26 showed that V5-MED22 colocalized with MED26 to the condensing chromatin and nucleolus in spermatocytes (Fig 2M–2P).

Consistent with the co-localization, V5-MED22 co-immunoprecipitated from testes extracts with other Mediator subunits, including MED26 and MED17 (S4 Fig). Although Med26 protein was still expressed in *Med22RNAi* testes (S2J Fig), localization of MED26 to chromatin and the nucleolus in spermatocytes was greatly reduced in early spermatocytes and was abolished in late spermatocytes by knockdown of Med22 by RNAi under control of Bam-Gal4 (S2G and S2H Fig, late spermatocytes marked by dashed triangles). Since localization but not protein accumulation of Med26 required function of Med22, localization of MED26 revealed by staining with anti-MED26 antibody was used below as an indirect readout of Med22 protein function in addition to detection of V5-MED22 by anti-V5 immunofluorescence.

While Bam-Gal4;UAS-Med22RNAi efficiently knocked down expression of Med22 mRNA (S2B Fig), MED22-3HA protein in testis extracts (S2I Fig) and V5-MED22 fusion protein specifically in spermatocytes (S2D and S2F Fig), knockdown of Med26 with the RNAi hairpin line tested was not effective (S2 Fig). Bam-Gal4;UAS-Med26RNAi did not affect expression of Med26 protein in testes or localization of Med26 protein in spermatocytes (S2K and S2L Fig), suggesting that the failure to cause a phenotype visible by phase contrast microscopy (S1 Table) was very likely due to inefficient knockdown of Med26.

### Localization of Mediator subunits to spermatocyte chromatin requires function of *aly* but not the tTAFs

**Fig 3:**
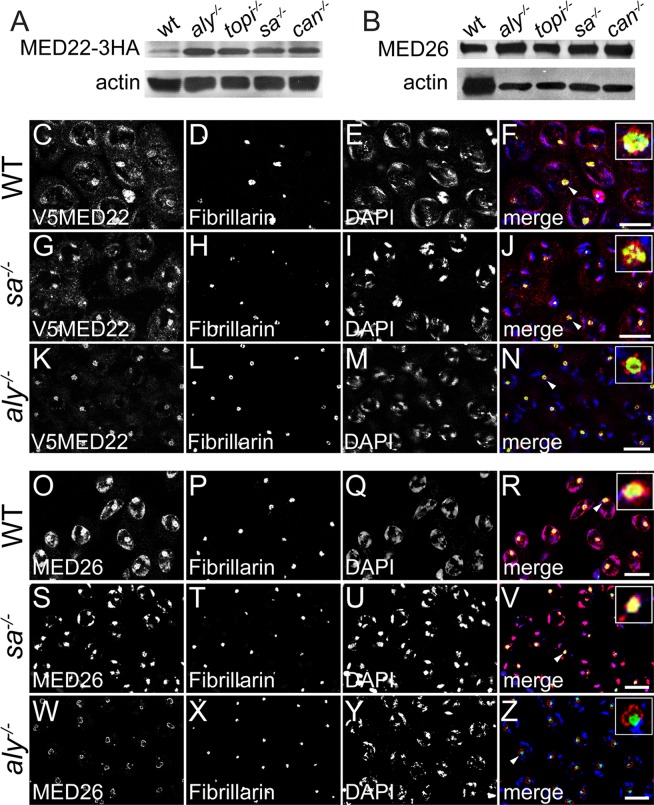
Localization of Mediator proteins to chromatin requires function of *aly* but not the tTAFs. **(A-B)** Western blots of wild type and indicated testes extracts probed with (A) anti-HA to detect MED22-3HA and (B) anti-MED26. Anti-actin: loading control. Crude extract of 30 pairs of testes loaded per lane. **(C-N)** Indirect immunofluorescence of (C-F) wild type (G-J) *sa* ^*-/-*^ and (K-N) *aly* ^*-/-*^ spermatocytes stained for (C, G and K) anti-V5 to detect V5-MED22, (D, H and L) Fibrillarin, (E, I and M) DAPI and (F, J and N) merge, red: V5-MED22, green: Fibrillarin, blue: DAPI. **(O-Z)** Indirect immunofluorescence of (O-R) wild type (S-V) *sa* ^*-/-*^ and (W-Z) *aly* ^*-/-*^ spermatocytes stained for (O, S and W) anti-MED26, (P, T and X) Fibrillarin, (Q, U and Y) DAPI and (R, V and Z) merge, red: anti-MED26, green: Fibrillarin, blue: DAPI. Insets in (F, J, N, R V and Z): close view of nucleoli marked by arrowheads. Bar: 10 μm.

Although MED22 and MED26 colocalized with the tTAFs in wild type spermatocytes, expression and localization of these mediator subunits to condensing chromatin and the nucleolus in spermatocytes did not require function of the tTAFs (Figs 3 and S5). Med22 protein, detected by probing western blots of testis extracts from flies carrying a rescuing genomic transgene encoding a MED22-3HA fusion (Materials and Methods) with anti-HA (Fig 3A) or MED26, detected with anti-MED26 (Fig 3B) was not reduced in testis extracts from flies mutant for the tTAFs, *sa* or *can*. (The higher levels of MED22-3HA and MED26 in all the meiotic arrest mutant samples were due to the accumulation of spermatocytes, in which the Mediator proteins are upregulated, in these samples.) Immunofluorescence staining with anti-V5 revealed localization of V5-MED22 to chromatin and the nucleolus in spermatocytes mutant for the tTAF *sa* (Fig 3G–3J) or the tTAF *can* (S5A–S5D Fig), as in wild-type (Fig 3C–3F). Similarly MED26 localized to the nucleolus and chromatin in spermatocytes in *sa* (Fig 3S–3V) or *can* mutant testes (S5E–S5H Fig), as in wild-type (Fig 3O–3R). In contrast, loss of function of the tMAC subunit *aly* strongly affected chromatin localization of MED22 and MED26, with chromatin staining of V5-MED22 and MED26 reduced to almost undetectable levels in *aly* mutant spermatocytes (Fig 3K–3N and 3W–3Z), although the MED22 and MED26 proteins were still expressed in testis from flies mutant for the tMAC subunits *aly* or *topi* (Fig 3A and 3B). Nucleolar localization of MED22 and MED26 was also different in *aly* mutant compared to wild type spermatocytes, as both proteins localized predominantly to perinucleolar loops surrounding the Fibrillarin domain (Fig 3N and 3Z), a pattern reminiscent of Sa protein localization in *aly* mutant spermatocytes [26].

### Localization of the tTAFs to chromatin in spermatocytes requires Mediator function

**Fig 4:**
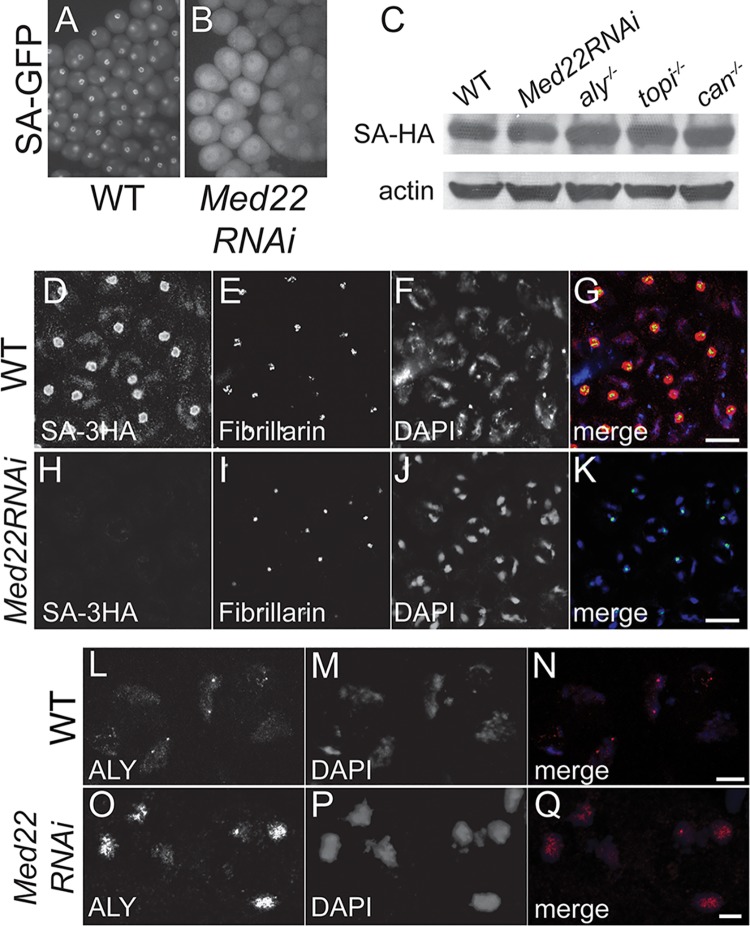
Localization of the tTAF protein Sa in spermatocyte nuclei depends on function of Med22. **(A and B)** live GFP squash showing SA-GFP expression and localization in (A) wild type and (B) *Med22RNAi* testes. **(C)** Western blot of wild type or indicated RNAi or mutant testes extracts showing protein levels of SA-3HA. Anti-actin: loading control. Crude extract of 30 pairs of testes loaded per lane. **(D-K)** Indirect immunofluorescence of (D-G) wild type and (H-K) *Med22RNAi* spermatocytes stained for (D and H) anti-HA to detect SA-3HA, (E and I) Fibrillarin, (F and J) DAPI and (G and K) merge, red: SA-3HA, green: Fibrillarin, blue: DAPI. Bars: 10 μm. **(L-Q)** Indirect immunofluorescence of (L-N) wild type and (O-Q) *Med22RNAi* spermatocytes stained for (L and O) Aly, (M and P) DAPI and (N and Q) merge, red: Aly, blue: DAPI. Bar: 2 μm.

Loss of function of Med22 in spermatocytes affected localization of the tTAF protein Sa (Figs 4 and S6). When SA-GFP encoded by a genomic rescue transgene [15] was viewed in unfixed squashed preparations (Fig 4A and 4B) of testes from either control or Bam-Gal4;UAS-Med22RNAi males, although much of the SA-GFP protein still appeared nuclear after loss of function of Med22, it was not enriched on either chromatin or in the nucleolus of *Med22RNAi* spermatocytes (Fig 4A and 4B). Similarly, either SA-GFP or SA-3HA expressed from an HA-tagged genomic rescue transgene [15], was not detected on chromatin or in the nucleolus by immunofluorescence staining for GFP or for the HA tag, respectively, in fixed spermatocytes in which Med22 function had been knocked down by RNAi (Figs 4D–4K and S6), even though Western blots of testis extracts showed that SA-3HA was expressed at levels comparable to wild type in *Med22RNAi* testes (Fig 4C). In contrast, the tMAC component Aly was still localized to the chromatin in *Med22RNAi* spermatocytes where it appeared more concentrated due to the highly condensed state of the chromatin in the mutant (Fig 4L–4Q). Together these data suggest that Med22 may act at a step between function of tMAC and recruitment of the tTAFs to chromatin.

### Med22 is required for expression of *aly* and *topi* dependent transcripts

**Fig 5:**
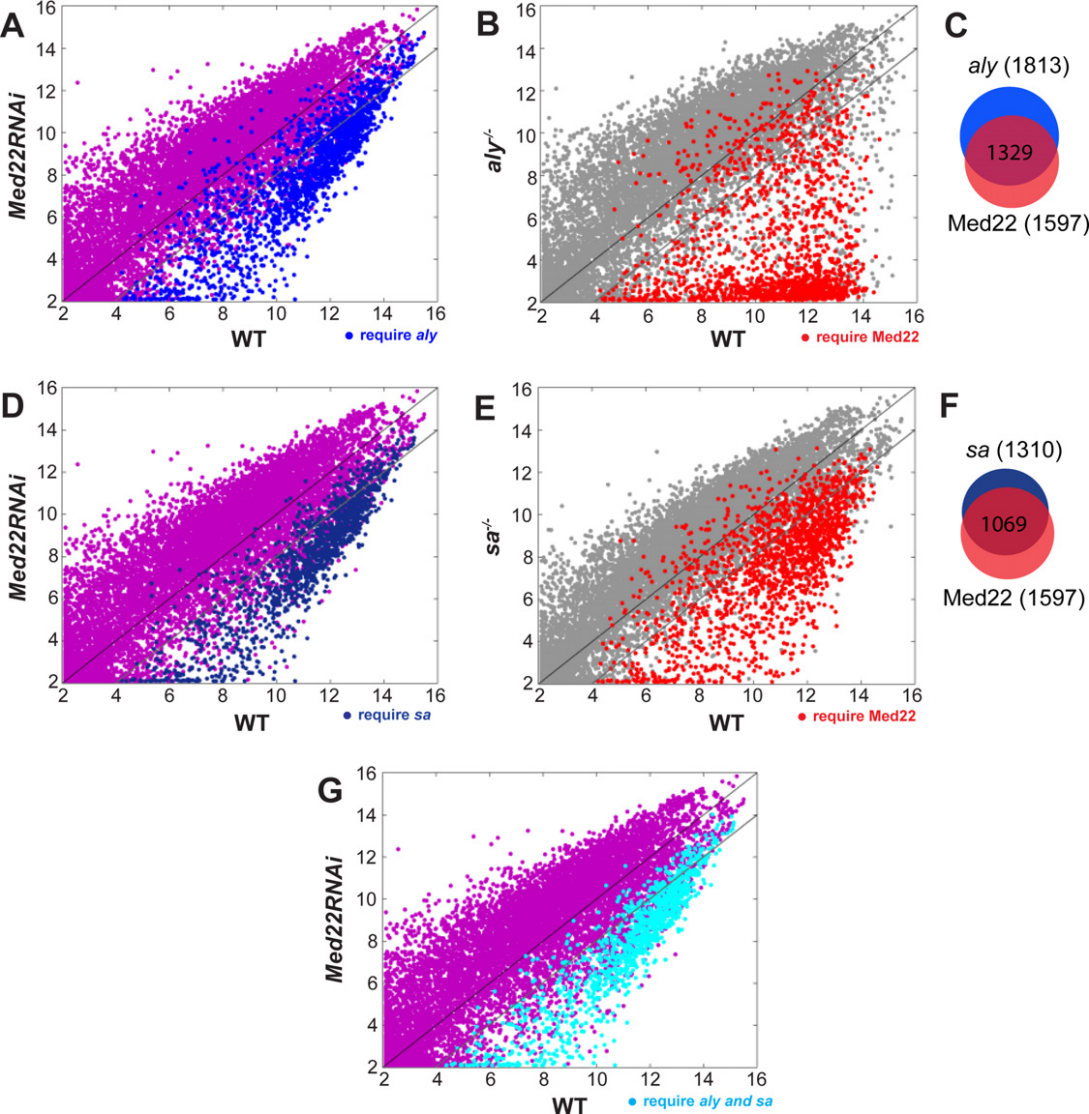
Med22 is required for expression of transcripts requiring tMAC and tTAF function. **(A, B, D, E and G)** Scatter plots comparing transcript levels in wild-type testes with (A, D and G) *Med22RNAi*, (B) *aly* ^*-/-*^, (E) *sa* ^*-/-*^. X and Y axes, log2 transformed gene expression values. (red dots in B and E) 1597 transcripts required function of Med22 for upregulation (≥2^2 fold higher in WT than in *Med22RNAi*); (blue dots in A) 1813 transcripts that required function of *aly* for upregulation (≥2^2 fold higher in WT than in *aly* ^*-/-*^); (blue dots in D) 1310 transcripts that required function of *sa* for upregulation (≥2^2 fold higher in WT than in *sa* ^*-/-*^); (cyan dots in G) 1022 transcripts that required function of both *aly* and *sa* (≥2^2 fold higher in WT than in *aly* ^*-/-*^ and in *sa* ^*-/-*^). FDR adjusted to P<0.05 for all samples. (C and F) Venn diagrams showing overlap of transcripts that required function of (C) *aly* or Med22 (1329 required both), (F) *sa* or Med22 (1069 required both).

The similar meiotic arrest phenotype and results from the analysis of protein localization in wild type and mutant testes suggest that Mediator may act in a pathway with tMAC and the tTAFs to activate expression of the transcription program for terminal differentiation in primary spermatocytes, with action of tMAC required for recruitment of Mediator to chromatin, and function of Mediator required for stable recruitment of the tTAFs to chromatin in spermatocytes. Consistent with this model, whole genome expression analysis of wild type versus *Med22RNAi* testes by microarray revealed that function of Med22 was required in spermatocytes for expression of many transcripts dependent on tMAC function (Fig 5). Indeed, most of the genes that required function of *aly* to be upregulated in testes also required function of Med22 to be upregulated (1329/1813 (73.3%) with a 4-fold cutoff) (Fig 5A and 5C). Reciprocally, a large fraction of the 1597 transcripts that required Med22 function to be upregulated in testes also required function of *aly* to be upregulated (1329/1597 (83.2%) with a 4-fold cutoff) (Fig 5B and 5C). Similarly, a major fraction of the 1310 transcripts that required *sa* for upregulation also required action of Med22 (1069/1310 (81.6%) with a 4-fold cutoff) (Fig 5D and 5F), and many of the transcripts that required function of Med22 in spermatocytes for upregulation in testes also required *sa* for upregulation (1069/1597 (66.9%) with a 4-fold cutoff) (Fig 5E and 5F). Consistent with the model above, the vast majority of the transcripts that required both *aly* and *sa* for upregulation also needed Med22 for upregulation (1022/1210 (84.5%) with a 4-fold cutoff) (Fig 5G).

**Fig 6:**
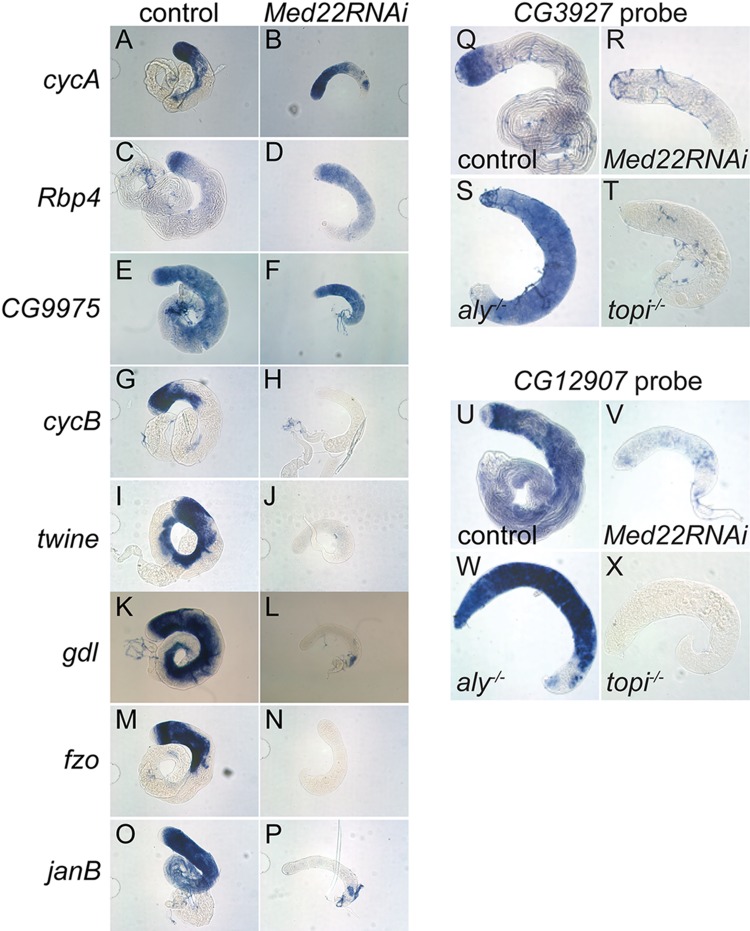
Similar transcript expression profiles between *Med22RNAi* and *topi*. **(A-P)** *in situ* hybridization to control and *Med22RNAi* knockdown testes processed in parallel in the same tube with antisense RNA probes for (A, B) *cycA*, (C, D) *Rbp4*, (E, F) *CG9975*, (G, H) *cycB*, (I, J) *twine*, (K, L) *gdl*, (M, N) *fzo* and (O, P) *janB*. **(Q-T)** *in situ* hybridization with antisense probe against the *topi*-dependent gene CG3927 in (Q) control, (R) *Med22RNAi*, (S) *aly* ^*-/-*^ and (T) *topi* ^*-/-*^ testes. **(U-X)** *in situ* hybridization with antisense probe against the *topi*-dependent gene CG12907 in (U) control, (V) *Med22RNAi*, (W) *aly* ^*-/-*^ and (X) *topi* ^*-/-*^ testes. Tip of testis to left in all panels.

Transcripts that required function of both *aly* and Med22 for cell type specific upregulation in spermatocytes included the core G2/M cell cycle regulators Cyclin B, *cdc2*/*twine* (Fig 6G–6J and Table 1) and its translational activator *boule* (Table 1), and spermatid differentiation factors such as *fzo* and *gdl*, for which full transcription also depend on function of the tTAFs (Fig 6K–6P and Table 1). Strikingly, action of Med22 was not required for cell type specific expression of a cohort of transcripts, represented by Cyclin A, *Rbp4* and *CG9975* (Fig 6A–6F), that are strongly activated in early spermatocytes but do not require function of tMAC or the tTAFs. Thus, action of Med22 is not required for activation of all genes newly expressed as male germ cells become spermatocytes, but rather appears to be mainly required for the cell type specific terminal differentiation transcription program controlled by tMAC and the tTAFs.

**Table 1:** Testis transcript expression profile of various genotypes by *in situ*.

genotype	WT	*aly*	tTAF	Med22	*topi*
transcripts					
general					
*cyclinA*	+	+	+	+	+
*CG9975*	+	+	+	+	+
*Rbp-4*	+	+	+	+	+
cell cycle					
*cycB*	+	-	+	-	-
*twine*	+	-	+	-	-
*Boule* ^a^	+	-	+	-	-
differentiation					
*fzo*	+	-	-	-	-
*janB*	+	-	-	-	-
*gdl*	+	-	-	-	-
*CG9173*	+	-	-	-	-
transcripts dependent on *topi* but not *aly*					
*CG3927*	+	+	+	-	-
*CG12907*	+	+	+	-	-

Allelic combinations used for each genotype: *y*.*w*, *aly* ^*2*^ */aly* ^*5P*^, *sa* ^*1*^ */sa* ^*2*^ and *can* ^*2*^ */can* ^*12*^, *Med22RNAi*, *topi* ^*Z0707*^ */topi* ^*Z2139*^.

(+) and (-) represent transcript expression On or Off, respectively, in spermatocytes of the designated mutant testis.

a: transcript expression analyzed by RT-PCR

Although both were found to be members of the tMAC complex, *aly* and *topi* mutants show slight differences in gene regulation: a small number of transcripts were shown to be expressed normally in *aly* mutant but not in *topi* mutant testes, including the transcripts of *CG3927* and *CG12907* [18]. Both the *CG3927* and *CG12907* transcripts were also not expressed in testes in which Med22 had been knocked down in spermatocytes by RNAi (Fig 6Q–6X and Table 1), suggesting that loss of function of Med22 may be more similar to loss of function of *topi* than to *aly*. Consistent with this, *Med22RNAi* and *topi* mutant testes also showed similar arrest phenotypes, which differ from the mutant phenotype of *aly* in terms of effect on spermatocyte chromatin (Fig 1E, 1E’, 1G, 1G’, 1H and 1H’).

### *topi* is required for localization of Mediator to chromatin in spermatocytes

**Fig 7:**
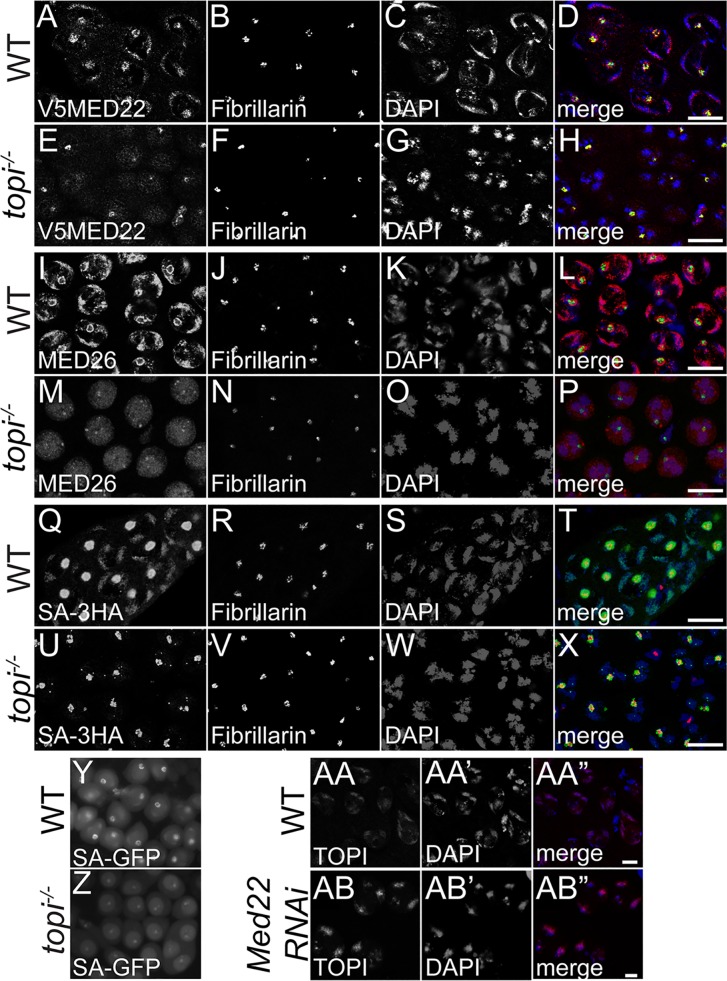
Localization of Mediator protein to spermatocyte chromatin requires *topi* function. **(A-H)** Indirect immunofluorescence of (A-D) wild type and (E-H) *topi* ^*-/-*^ spermatocytes stained for (A and E) anti-V5 to detect V5-MED22, (B and F) Fibrillarin, (C and G) DAPI and (D and H) merge, red: V5-MED22, green: Fibrillarin, blue: DAPI. Bars: 10 μm. **(I-P)** Indirect immunofluorescence of (I-L) wild type and (M-P) *topi* ^*-/-*^ spermatocytes stained for (I and M) anti-MED26, (J and N) Fibrillarin, (K and O) DAPI and (L and P) merge, red: anti-MED26, green: Fibrillarin, blue: DAPI. Bars: 10 μm. **(Q-X)** Indirect immunofluorescence of (Q-T) wild type and (U-X) *topi* ^*-/-*^ spermatocytes stained for (Q and U) anti-HA to setect SA-3HA, (R and V) Fibrillarin, (S and W) DAPI and (T and X) merge, red: SA-3HA, green: Fibrillarin, blue: DAPI. Bars: 10 μm. **(Y and Z)** live GFP squash showing SA-GFP expression and localization in (Y) wild type and (Z) *Med22RNAi* testes. **(AA-AB”)** Indirect immunofluorescence of (AA-AA”) wild type and (AB-AB”) *Med22RNAi* spermatocytes stained for (AA and AB) Topi, (AA’ and AB’) DAPI and (AA” and AB”) merge, red: Topi, blue: DAPI. Bars: 2 μm.

Even though MED22-3HA protein accumulated to even higher levels (Fig 3A) in *topi* null mutant versus WT testes extracts (due to accumulation of the arrested spermatocytes in *topi* mutant testes), MED22-3HA failed to localize properly to chromatin in *topi* mutant testes (Fig 7A–7H). Similarly, Med26 protein was expressed in *topi* mutant testes (Fig 3B), localization of MED26 protein was strongly affected in spermatocytes mutant for *topi*. Although the majority of the MED26 immunofluorescence signal detected was nuclear, in contrast to in wildtype, MED26 did not colocalize with the bivalent chromosomes in *topi* mutant spermatocytes. (Fig 7I–7P). Consistent with Med22 being required for localization of the tTAFs, Sa protein also failed to localize to the chromatin in *topi* mutant spermatocytes, shown either by anti-HA staining of testes from flies expressing the SA-3HA transgene (Fig 7Q–7X) or by imaging fluorescence of GFP in unfixed squashed preparations of testes from flies carrying a *sa-GFP* genomic rescue transgene (Fig 7Y and 7Z).

**Fig 8:**
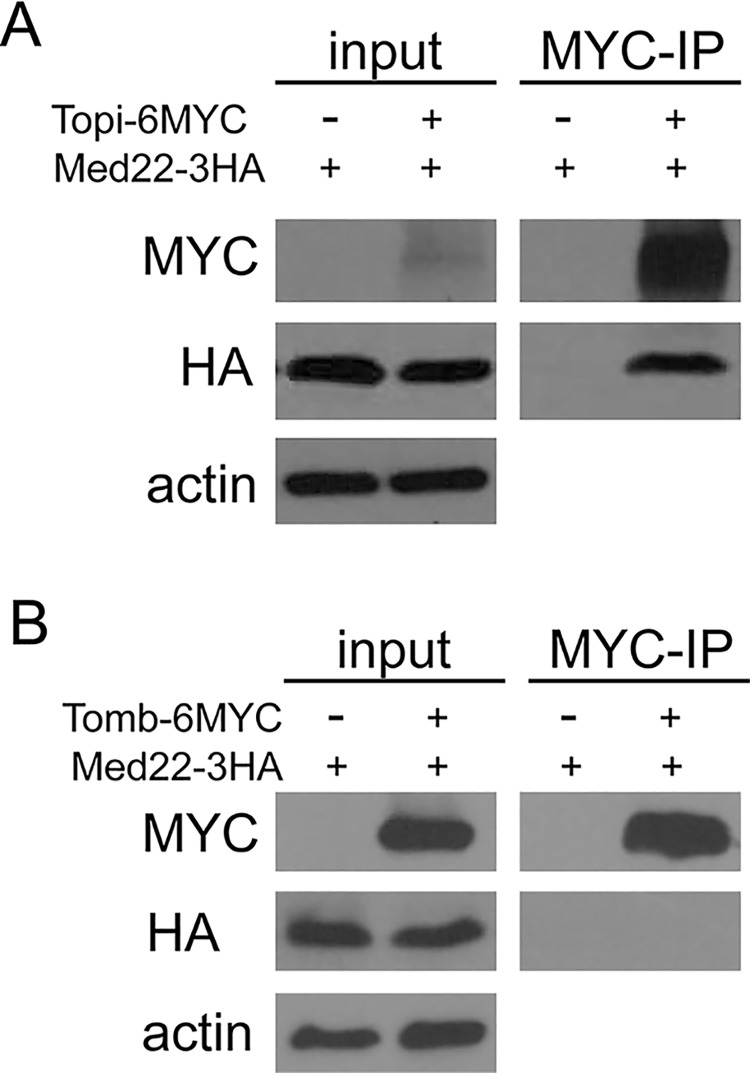
MED22 and Topi physically interact when co-expressed in S2 cells. **(A)** MED22-3HA co-immunoprecipitated with TOPI-6MYC. S2 cell extracts coexpressing MED22-3HA with or without TOPI-6MYC immunoprecipitated with anti-MYC and blotted with anti-HA. Input is 1/10 of each pre-immunoprecipitation crude cell extract. Anti-actin blot served as input loading control to show equal amount of crude cell extract was used for the two samples. **(B)** MED22-3HA failed to co-immunoprecipitate with TOMB-6MYC. S2 cell extracts coexpressing MED22-3HA with or without TOMB-6MYC immunoprecipitated with anti-MYC and blotted with anti-HA. Input is 1/10 of each pre-immunoprecipitation crude cell extract. Anti-actin blot served as input loading control to show equal amount of crude cell extract was used for the two samples.

In contrast, localization of *topi* protein to chromatin in spermatocytes did not require Med22 (Fig 7AA–7AB”), suggesting that *topi* acts upstream of Mediator in the recruitment pathway. These results, together with the similar effects on gene expression of Med22 knockdown in spermatocytes and of loss of function of *topi*, raised the possibility that function of predicted sequence specific DNA binding components of tMAC, such as Topi, may be involved in recruiting Mediator to target loci. Consistent with this idea, immunoprecipitation of Myc-tagged Topi brought down HA-tagged Med22 when the two proteins were coexpressed in S2 cells (Fig 8A). In contrast, the core tMAC component Tombola failed to co-immunoprecipitate Med22 under similar conditions (Fig 8B). These data altogether suggest that interaction of Mediator with tMAC, mediated at least partially through binding of Med22 to Topi, serves a crucial step in establishing the cascade of transcriptional activation events in differentiating spermatocytes.

## Discussion

Activation of cell-type-specific gene expression profiles underlies terminal differentiation programs in both embryonic and adult stem cell lineages. In many cases such stage- and cell-type-specific gene expression programs require cooperative action of sequence-specific transcriptional activators and tissue-specific components of the basal transcription machinery [26,33,34]. We have previously demonstrated that full activation in spermatocytes of the transcription program for terminal differentiation of male gametes requires sequential action of the testis specific tMAC complex and five testis specific homologues of components of the general transcription factor complex TFIID (the tTAFs) [26]. How tMAC and the tTAFs function at promoters of differentiation genes is not yet understood at the molecular level although function of tMAC was needed to recruit the tTAF protein Sa to promoters of spermatid differentiation genes [26]. Our new data presented here indicate that subunits of the Mediator coactivator complex mediate the regulatory function of tMAC on the tTAFs, acting in a pathway to turn on robust expression of terminal differentiation genes in spermatocytes.

Potentially functioning as a testis specific TFIID complex, the tTAFs are needed for full activation of transcription of hundreds of spermatid differentiation genes in primary spermatocytes but are dispensable for transcription of meiotic cell cycle genes activated in the same cells (Table I and [14,15]). It is possible that the gene selectivity of the tTAFs is determined primarily by structures of the promoters of differentiation genes. The general TAFs and TFIID were shown to confer promoter selectivity and facilitate transcription activation at promoters with no or less stringent TATA boxes in *Drosophila* systems [35,36]. Alternatively, as our results now suggest, the gene selectivity of the tTAFs may be partly achieved through DNA sequence specific components in the tMAC complex. tMAC contains at least two potential sequence-specific DNA-binding proteins, Tomb and Topi [18,20]. We found that Topi and MED22 physically interact when coexpressed in S2 cells (Fig 8). The tTAF protein Sa failed to localize to meiotic chromatin without Med22 function, suggesting the tTAFs may be recruited or stabilized by Mediator at promoters of the spermatid differentiation genes. Similar coactivator cross-talk between Mediator and canonical TAFs was observed during activation of the metal response gene, MtnA, in *Drosophila cell* culture [37]. Although TFIID and Mediator were recruited separately to the MtnA promoter, TFIID was only functional in the presence of Mediator [37].

Although expression and subcellular localization of Med22 protein proceeded protein accumulation and localization of Sa in spermatocytes, and the correct localization of Sa relied on function of Med22, our data do not prove direct recruitment of Sa by Med22. The tTAFs, including Sa, may be recruited by other mechanisms to promoters of differentiation genes, with function of Mediator needed to stabilize and facilitate assembly and function of the tTAF containing preinitiation complex. Despite that Sa and Mediator colocalized to both chromatin and the nucleolus in spermatocytes based on immunofluorescence staining, Sa protein was predominantly detected at the nucleolus (Fig 4), whereas the Mediator signal was more evenly distributed between chromatin and the nucleolus (Figs 2 and S3), again suggesting that Sa may not be directly recruited by Mediator, at least to the nucleolus. It is possible that once localized, Sa is stabilized through interaction with Mediator since both the nucleolar and chromatin localization of Sa was completely abolished in *Med22RNAi* (Fig 4). We were not able to detect direct interaction between Mediator subunits and tTAF components in testis extracts. However, considering the limitation of the *Drosophila in vivo* system for such biochemical assays, we cannot rule out the possibility that the two complexes physically interact in spermatocytes, since interactions between Mediator and TFIID have been demonstrated in vitro [38].

Of all the Mediator subunits we attempted to knock down in spermatocytes by RNAi, knock downs of only a few caused the meiotic arrest phenotype. Of these, *Med22RNAi* is the only knockdown for which the meiotic chromatin resembled tTAF mutant chromatin. Phenotypic variations among different Mediator subunits were also observed in a previous study in SL2 cells, in which each of mediator subunit was knocked down by RNAi [39]. The heterogeneity of Mediator subunit knockdown phenotypes may be a result of variation in RNAi efficiency. Indeed, analysis of protein by immunofluorescence revealed that the RNAi hairpin against Med26 we tested did not knock down expression of the Med26 protein (S2 Fig). Since we use the Gal4-UAS system to drive RNA hairpin expression in spermatocytes, it is worth noting that Mediator had been shown to be required for Gal4 mediated transcription at UAS-containing promoters [40]. Therefore, Mediator subunits needed for structural integrity of the complex or directly involved in interaction with Gal4-AD might not give strong phenotypes due to the potential negative feedback on the RNAi process. Alternatively, the differences in RNAi knockdown phenotypes of individual subunits could reflect functional diversity of particular subunits.

Although previously thought to be a generally needed inert protein complex which passively bridge interactions between transcription activators and the general transcription machinery, including TFIID and PolII, recent studies have revealed a more active and even gene selective role for Mediator in transcription activation. Many Mediator subunits were found to selectively modulate and integrate regulatory signals of specific cellular and metabolic pathways [41,42,43,44,45,46,47]. Previous studies also showed several Mediator subunits functioned as adaptors to bridge interactions of particular transcription factor(s) to the Mediator complex or subcomplexes. For example, the C-terminal domain and the activation domain of p53 specifically bind to the MED1 and MED17 subunits of Mediator, respectively [48,49]. MED15 binds strongly to the activation domain of SREBP-1α to regulate lipid homeostasis [43], and MED23 binding to the ELK-1 activation domain is required specifically for adipogenesis [41]. More interestingly, MED14 and MED25 mutants had opposite effects on cell size control in *Arabidopsis*, suggesting distinct Mediator subunits coexisting in the same cells can have distinct mechanistic roles in transcription regulation [42,50]. Our work suggests Mediator is recruited in primary spermatocytes to target genes by the tissue- and cell type-specific transcription factor Topi through its physical interaction with MED22. Importantly, consistent with being recruited by sequence specific activators in the tMAC, Mediator is not generally required in spermatocytes for activating transcription, as most genes which did not require function of tMAC (as seen with *aly* ^*-/-*^) or tTAFs (as seen with *sa* ^*-/-*^), were expressed at relatively normal levels in *Med22RNAi* testes (Fig 5 and Table 1).

Although we only detected physical interaction between MED22 and Topi (Fig 8), it is certainly possible that other putative sequence specific DNA binding factors in tMAC, such as Tomb, or peripheral to tMAC, such as Achi/Vis also recruit Mediator through specific interactions with other Mediator subunits. Topi and Achi/Vis were suggested to be both needed at promoters of most genes controlled by the *aly* class of meiotic arrest genes [20]. In spermatocytes, signals from different components of tMAC instructing activation of transcription may be integrated by Mediator through interactions between tMAC subunits and specific Mediator subunits, before being transduced to the general transcription machinery containing the tTAFs. Our study on how transcriptional activation signals maybe routed through Mediator to the general machinery in differentiating spermatocytes will also shed light on how master transcription factors and Mediator-enriched super-enhancers [5] may interact with the basal machinery in a gene selective fashion.

## Materials and Methods

### Fly husbandry

*Drosophila* stocks were maintained on cornmeal and dextrose media at 22°C. Crosses and experimental flies were grown on cornmeal and molasses media at 25°C. All markers used were previously described [51](www.flybase.org). Fly strains were obtained from the Bloomington Stock Center. Flies carrying RNAi hairpins were from the Vienna *Drosophila* RNAi Center (VDRC). tMAC and tTAF mutant strains used were *aly* ^*2*^/*aly* ^*5p*^, *topi* ^*Z0707*^/*topi* ^*Z2139*,^ *can* ^*2*^ */can* ^*12*^ or sa^*1*^ /sa^*2*^ trans-heterozygotes. Wild-type control flies were *y* ^*1*^,*w* ^*1118*^ unless otherwise stated. SA-GFP and SA-3HA flies both contain all the sequences necessary to rescue *sa* null mutants and are expressed specifically in spermatocytes like the endogenous protein [15].

### Spermatocyte specific RNAi against Mediator subunits

Virgin UAS-Dicer2;;Bam-Gal4 females were crossed to *y*,*w* males (control) or males carrying RNAi-hairpin against each of the Mediator subunits and grown at 25°C. Testes from resulting F1 males were dissected for further analysis. RNAi hairpins reported in this work are VDRC#104581(Med22), VDRC#51476(Med26) and those listed in S1 Table. For comparing SA-GFP, SA-3HA, V5-Med22 or Med22-3HA expression in Mediator RNAi knockdown testes, crosses were set up so experimental flies and sibling controls always had the same copies of the relevant transgene.

The Med22 RNAi hairpin from VDRC (#104581) contained only 136 base pairs of cDNA sequences near the C-terminus of the gene and had no predicted off-targets. We designed two additional UAS-hairpins containing a 249 bp sequences from the remaining Med22 N-terminal cDNA, in opposite orientation, following a previously published protocol[52]. However, neither of the newly developed hairpins knocked down Med22 expression nor produced any phenotype. Primers used to amplify the 249 base pair cDNA sequences were 5’-GACAACCATTTTGCCCCAG and 5’-AGGTACTGTTTTAGGTCGG.

### Generation of Med22 and Topi tagged constructs

mCherryV5-Med22 and Med22-3HA: An EcoRI site was introduced right before the start ATG of Med22 genomic coding sequences. V5 sequences were added onto mCherry by PCR with a mCherry reverse primer containing the V5 sequences. The mCherryV5 sequences were inserted in frame into the EcoRI site to creat a mCherryV5-Med22 N terminal fusion. The entire coding sequences were then fused in between Med22 endogenous promoter and 3’ sequences before inserting into pCaSpeR4 [53]. The mCherryV5-Med27 construct was created similarly. Med22-3HA transgene was constructed similarly except that a XhoI site was introduced immediately before the stop codon and used to insert PCR amplified 3HA sequences. The resulting constructs were introduced into flies via P-element mediated transformation. Both transgenes fully rescued the lethality of the Med22 null alleles (see below) demonstrating functionality of the tagged proteins.

UAST-Med22-3HA and UAST-Topi-6MYC: 3HA or 6MYC sequences were PCR amplified, fused immediate before the stop codon and in frame to Med22 or *topi* cDNA, respectively. The resulting fusion cDNAs were inserted into EcoRI-XbaI sites of the pUAST vector [54]. UAST-Tomb-6MYC: 6MYC sequences were PCR amplified, fused immediate before stop codon and in frame to *Tomb* cDNA. The resulting fusion cDNA was inserted into EagI-XbaI sites of the pUAST vector.

### Med22 deletion alleles using CRISPR (clustered regularly interspaced short palindromic repeats)

Seed sequences of three CRISPR guide RNAs targeting the first exon of Med22 were selected using Zhang lab web tool (http://www.genome-engineering.org/crispr): Med22_CRISPR_01 AGAAGCGCCTCCTTCGACTG; Med22_CRISPR_02 AATGGTTGTCCTGGATCCGC; Med22_CRISPR_03 GTCCTATAATGCGCGCCTCA. Templates for each Guide RNA were PCR amplified with a universal reverse primer containing the single guide RNA (sgRNA) sequence for incorporation into the Cas9 enzyme (AAAAGCACCGACTCGGTGCCACTTTTTCAAGTTGATAACGGACTAGCCTTATTTTAACTTGCTATTTCTAGCTCTAAAAC) and a specific forward primer containing [T7 promoter] -[CRISPR seed sequences]-[complementary sequences to the universal primer] ([GAAATTAATACGACTCACTATA][CRISPR seed sequences][GTTTTAGAGCTAGAAATAGC]) as Bassett et al. described [55]. sgRNAs were in vitro transcribed with MEGAshortscript T7 kit (Life Technologies). After in vitro transcription, the reaction mixture was Turbo DNase (Life Technologies) digested for 15 minutes at 37°C followed by phenol-chloroform extraction and ethanol precipitation. A mixture of the three sgRNAs, each at 800ng/μL was injected into [nos-Cas9]attP2 embryos[56] to create deletions in the Med22 locus.

To select for lethal Med22 deletion alleles on the X chromosome, female adult flies that developed from injected embryos were crossed to *w* ^*-*^;MKRS/TM6B flies (the balancers were used to follow and separate out the Cas9 expressing cassette on chromosome III). 20 individual F1 females from each cross were then mated with FM7i, P[ActGFP]JMR3 males and allowed to lay eggs for 1 week before applied to PCR analysis to detect genomic deletions with these primers: Med22-Crispr-F-AGTTAGGACGGTATTTATGG and Med22-Crispr-R-GCCAGCTTGAGTATTTCTGG. 8 individual FM7 balanced F2 females from either F1 mothers which carry detectable deletions in the PCR fragment or F1 mothers which failed to produce *w* ^*+*^ male progenies were again crossed to FM7i, P[ActGFP]JMR3 males to select for balanced Med22 lethal deletion stocks. PCR fragment was amplified from the mutation baring chromosome using the above Med22-Crispr primers and sequenced.

Two lethal alleles were used in this study. *Med22_Crispr* ^*1*^ has a 34 base pair deletion (deleted: GCGGATCCAGGACAACCATTTTGCCCCAGTCGAA) after the second codon and produces 4 more frame shifted amino acids before stop. *Med22_Crispr* ^*2*^ has a single base pair deletion (deleted: G) in codon 3 to cause a frame shift to produce 13 more amino acids before stop. The lethality of both alleles was rescued with either the mCherryV5-Med22 or the Med22-3HA transgenes. The rescued hemizygous males were fertile.

### Western blotting

Testes were dissected in cold PBS containing Cocktail Protease Inhibitors (Roche), transferred to 50μl cold PBS containing 1xSDS sample buffer before flash frozen in liquid nitrogen and stored in -80°C. Collected samples were homogenized with a blue pestle, boiled for 10 minutes, and spun for 2 minutes before loaded onto a 10% SDS-PAGE gel (Bio-Rad). Proteins were transferred onto a PVDF membrane (Bio-Rad) and blocked in blocking solution (1xTBS+ 0.1% Tween-20+ 5% non-fat milk). Primary and HRP-conjugated secondary antibodies were diluted in blocking solution and added to the membrane to incubate overnight at 4°C. Signals were detected with WESTERN LIGHTNING Plus-ECL (PerkinElmer) and exposed on Kodak BIOMAX XAR X-ray film. Membranes were washed extensively before blotted again with anti-actin (clone C4, Millipore) as loading control.

### Co-immunoprecipitation in S2 cell and testes exacts

S2 cells were co-transfected with plasmids for the driver Act-Gal4 and UAST expression, UAST-Med22-3HA and UAST-Topi-6MYC or UAST-Med22-3HA and UAST-Tomb-6MYC with Effectene reagent (Qiagen). 200 pairs of testes were dissected from either V5-MED22 or *y*.*w*. males for anti-V5 co-immunoprecipitation. Co-immunoprecipitation was performed following a previously published protocol [57]. Antibodies: anti-MYC (Millipore), anti-HA (Abcam), anti-V5 (Invitrogen), anti-MED26 and anti-MED17 [37].

### Microscopy and immunofluorescence

For live phase/Hoechst squash, dissected testes were cut open on a slide in 70 μl of PBS containing 2μg/ml Hoechst 33342, gently squashed by lowering a coverslip and wicking out some solution, and examined immediately on a Zeiss Axioplan microscope. Images were taken with a Photometrics COOLSNAP EZ camera and processed with Adobe Photoshop software.

Immunofluorescence was performed as previously described [15], using the following antibodies: mouse anti-GFP (Roche), rabbit anti-HA (Bethyl Laboratories), mouse anti-V5 (Invitrogen), mouse anti-MYC (Millipore), anti-Aly [22], anti-Topi [20], mouse anti-Fibrillarin (Cytoskeleton), rabbit anti-Fibrillarin (Abcam) and anti-Med26 [37]. Alex-fluor-conjugated goat secondary antibodies were used. Images were captured with either a Leica SP2 confocal microscope or a Leica SP5 confocol microscope and processed with Adobe Photoshop software.

### Microarray and RNA *in situ* hybridization

Affymetrix 2.0 *Drosophila* chips were processed by the Stanford PAN facility (pan.stanford.edu). Data set for three biological replicates of *Med22RNAi* were prepared and analyzed with the published *red*,*e*, *aly* and *sa* data sets as previously described [27].

RNA *in situ* hybridization was performed following previously described protocol [58]. RNA probes for the representative genes tested were previously described [27] except for Med22. To generate probe for Med22, full length cDNA of Med22 was subcloned into pBluescript-SK^+^. The resulting plasmid was linearized as a template for labeling with DIG-labeling reagents (Roche) following manufacture’s instruction. CG12907 and CG3927 probes were in vitro transcribed using a PCR amplified cDNA fragment with a T7 promoter sequences introduced from the reverse primer as previously described[20]. RNA probes over the size of 200 bp were hydrolyzed.

## Supporting Information

S1 Fig Effects of Mediator RNAi inversely correlate with Levels of Boule protein accumulation. **(A and B)** Western blots of testis extracts of wild type and RNAi knockdown of each of the Mediator subunits indicated, probed with anti-Boule. Anti-actin: loading control. Crude extract of 30 pairs of testes loaded per lane.
(TIF)

S2 Fig *Med22RNAi* Driven by Bam-Gal4 specifically and effectively knock down Med22 expression in spermatocytes. **(A and B)** *in situ* hybridization with Med22 gene specific anti-sense probe showed (A) Med22 transcript expression in control RNAi testis and (B) lowered Med22 transcript expression in *Med22RNAi* knock down testis. Bracket: Med22 transcript enriched in early spermatocyte stages in wild type. **(C-F)** Indirect immunofluorescence of V5-MED22 expression in (C) apical region of control versus (D) apical region of *Med22RNAi* testis, (E) control spermatocytes and (F) *Med22RNAi* spermatocytes. Expression of V5-MED22 remain unaffected by *Med22RNAi* in apical region where the Bam-Gal4 expression driver is not active so the *Med22RNAi* hairpin is not expressed. Arrowheads: nuclei of somatic cyst cells, in which the Bam-Gal4 driver is not active. Asterisks: tip of testis. **(G and H)** Indirect immunofluorescence of anti-MED26 staining in (G) control RNAi versus (H) *Med22RNAi* spermatocytes. Dashed triangles in (H): late spermatocytes with completely abolished anti-MED26 signal by *Med22RNAi* **(I and J)** Western blots of control and *Med22RNAi* testis extracts showing (I) MED22-3HA and (J) MED26 protein levels. Crude extract of 30 pairs of testes loaded per lane. **(K and L)** Indirect immunofluorescence of anti-MED26 staining in (G) control RNAi and (H) *Med26RNAi* spermatocytes. Bars: in (D) 25 μm, in (F, H and L) 10 μm. Knockdown of Med22 by RNAi abolished detection of V5-Med22 on chromatin in spermatocytes. However, low level staining of the nucleolus by anti-V5 remained, indicating either background crossreactivity of the anti V5 with a nucleolar epitope, or some residual V5-Med22 protein remaining due to incomplete knockdown or perdurance from the spermatogonial stages.
(TIF)

S3 Fig Mediator proteins localize to chromatin and the nucleolus in spermatocytes. **(A-C)** Indirect immunofluorescence of testis showing elevated levels of MED26 accumulation in spermatocytes (A) anti-MED26, (B) anti-Fibrillarin, (C) merge, red: MED26, green: Fibrillarin. Asterisks: tip of testis. Bar: 25 μm. **(D-F)** V5-MED27 colocalize with SA-3HA in spermatocytes. (D) anti-V5 to detect V5-MED27, (E) anti-HA to detect SA-3HA, (F) merge, red: V5-MED27, green: SA-3HA. Asterisks: tip of testis. Bar: 10 μm. **(G-K)** merged images of MED26 (green) and Fibrillarin (red) showing MED26 protein enriches and gradually concentrates onto meiotic chromatin and nucleolus as spermatocyte develop from (G) young through (K) more mature stages. Bars: 4 μm.
(TIF)

S4 Fig V5-Med22 co-immunoprecipitates with MED26 and MED17. Testis extracts of V5-MED22 or y.w. were immunoprecipitated with anti-V5 and blotted with anti-MED26 or anti-MED17. 200 pairs of testis used per IP. Input is 1/10 of each pre-immunoprecipitation crude cell extract. V5-MED22 was not visible by anti-V5 blot from the V5-MED22 input (crude extract from equivalent of ~20 pairs of V5-MED22 testes) but was visible by anti-V5 blot after anti-V5 IP.
(TIF)

S5 Fig Mediator proteins still localize to chromatin in *can* mutant spermatocytes. **(A-H)** Indirect immunofluorescence of *can* ^*-/-*^ spermatocytes stained for (A) anti-V5 to detect V5-MED22, (E) anti-MED26, (B and F) Fibrillarin, (C and G) DAPI and (D and H) merge, red: in (D) V5-MED22, in (H) anti-MED26, green: Fibrillarin, blue: DAPI. Bars: 10 μm.
(TIF)

S6 Fig Localization of SA-GFP in spermatocyte nuclei requires function of Med22 (A-H) Indirect immunofluorescence of *(A-D)* wild type and (E-H) *Med22RNAi* testis stained for (A and E) anti-GFP to detect Sa-GFP, (B and F) Fibrillarin, (C and G) DAPI and (D and H) merge, green: SA-GFP, red: Fibrillarin, blue: DAPI. Asterisks: tip of testis. Bars: 50 μm.
(TIF)

S1 Table Summary of Mediator RNAi phenotypes. (PDF)
