# Supplementary figures and images for: Recruitment of Mediator Complex by Cell Type and Stage-Specific Factors Required for Tissue-Specific TAF Dependent Gene Activation in an Adult Stem Cell Lineage
Source: PLoS Genet. 2015 Dec 1;11(12):e1005701. doi: 10.1371/journal.pgen.1005701 (PMC4666660; doi:10.1371/journal.pgen.1005701)

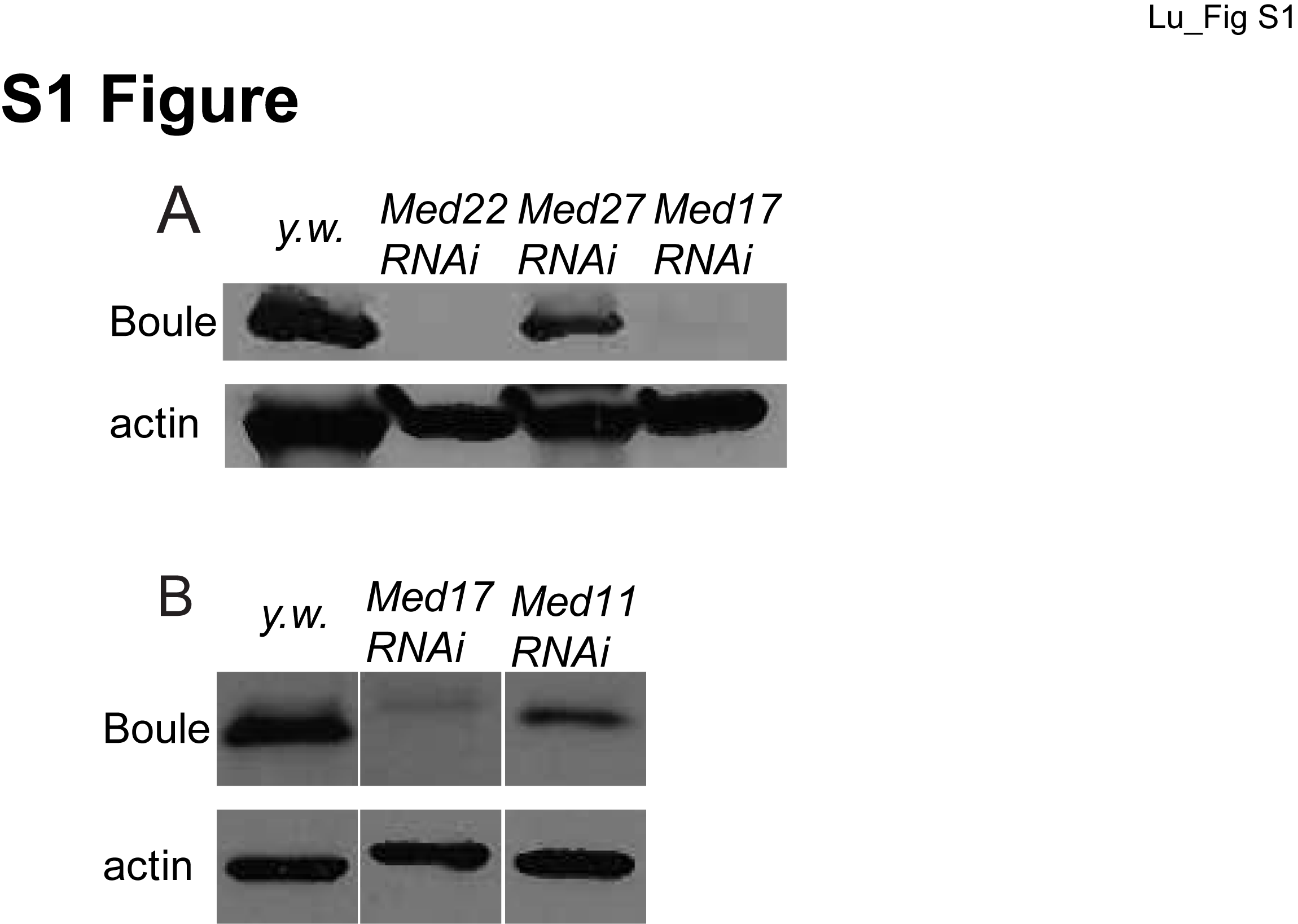

Supplement: S1 Fig — (A and B) Western blots of testis extracts of wild type and RNAi knockdown of each of the Mediator subunits indicated, probed with anti-Boule. Anti-actin: loading control. Crude extract of 30 pairs of testes loaded per lane. (TIF) [file pgen.1005701.s001.tif]

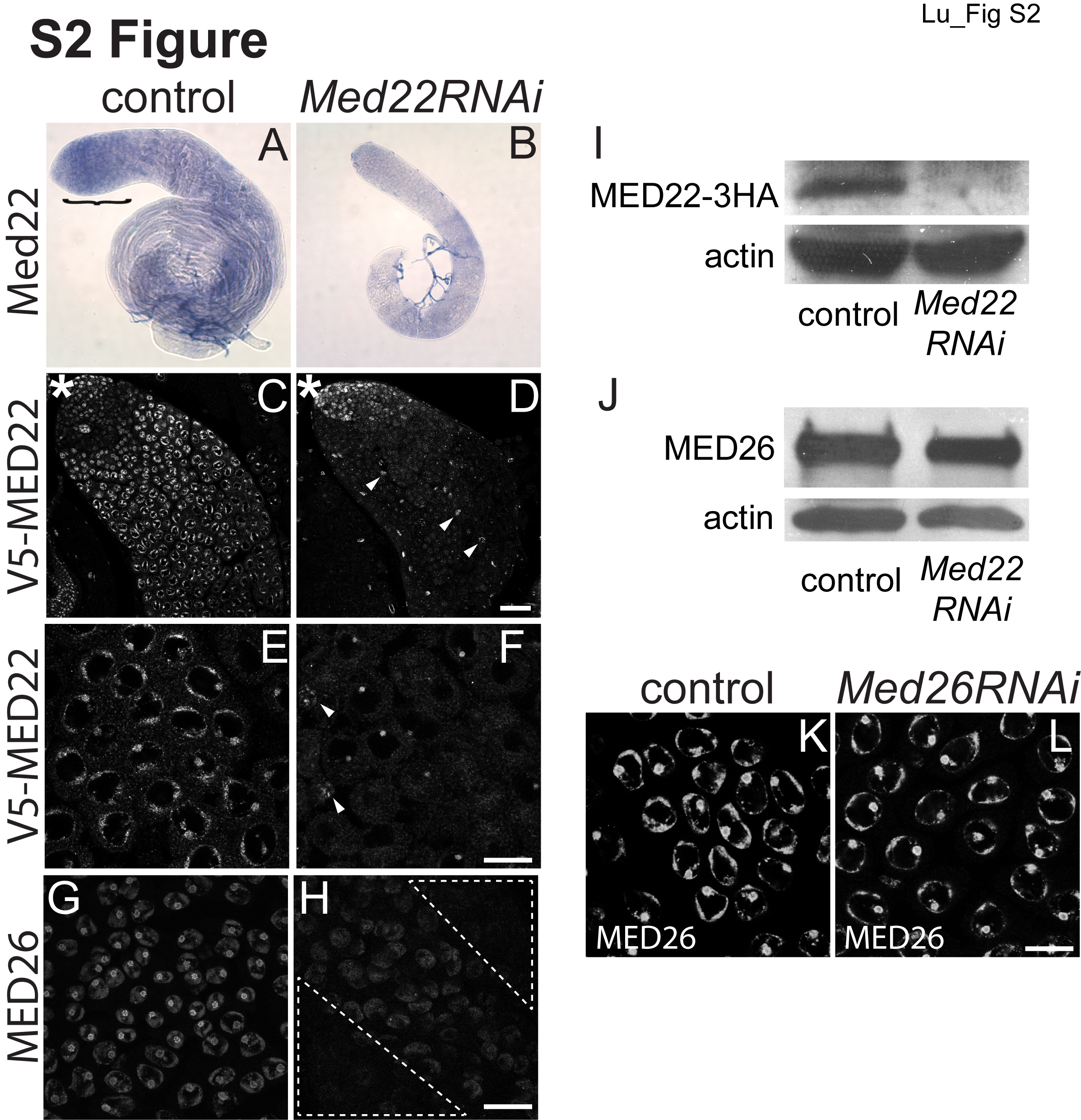

Supplement: S2 Fig — (A and B) in situ hybridization with Med22 gene specific anti-sense probe showed (A) Med22 transcript expression in control RNAi testis and (B) lowered Med22 transcript expression in Med22RNAi knock down testis. Bracket: Med22 transcript enriched in early spermatocyte stages in wild type. (C-F) Indirect immunofluorescence of V5-MED22 expression in (C) apical region of control versus (D) apical region of Med22RNAi testis, (E) control spermatocytes and (F) Med22RNAi spermatocytes. Expression of V5-MED22 remain unaffected by Med22RNAi in apical region where the Bam-Gal4 expression driver is not active so the Med22RNAi hairpin is not expressed. Arrowheads: nuclei of somatic cyst cells, in which the Bam-Gal4 driver is not active. Asterisks: tip of testis. (G and H) Indirect immunofluorescence of anti-MED26 staining in (G) control RNAi versus (H) Med22RNAi spermatocytes. Dashed triangles in (H): late spermatocytes with completely abolished anti-MED26 signal by Med22RNAi (I and J) Western blots of control and Med22RNAi testis extracts showing (I) MED22-3HA and (J) MED26 protein levels. Crude extract of 30 pairs of testes loaded per lane. (K and L) Indirect immunofluorescence of anti-MED26 staining in (G) control RNAi and (H) Med26RNAi spermatocytes. Bars: in (D) 25 μm, in (F, H and L) 10 μm. Knockdown of Med22 by RNAi abolished detection of V5-Med22 on chromatin in spermatocytes. However, low level staining of the nucleolus by anti-V5 remained, indicating either background crossreactivity of the anti V5 with a nucleolar epitope, or some residual V5-Med22 protein remaining due to incomplete knockdown or perdurance from the spermatogonial stages. (TIF) [file pgen.1005701.s002.tif]

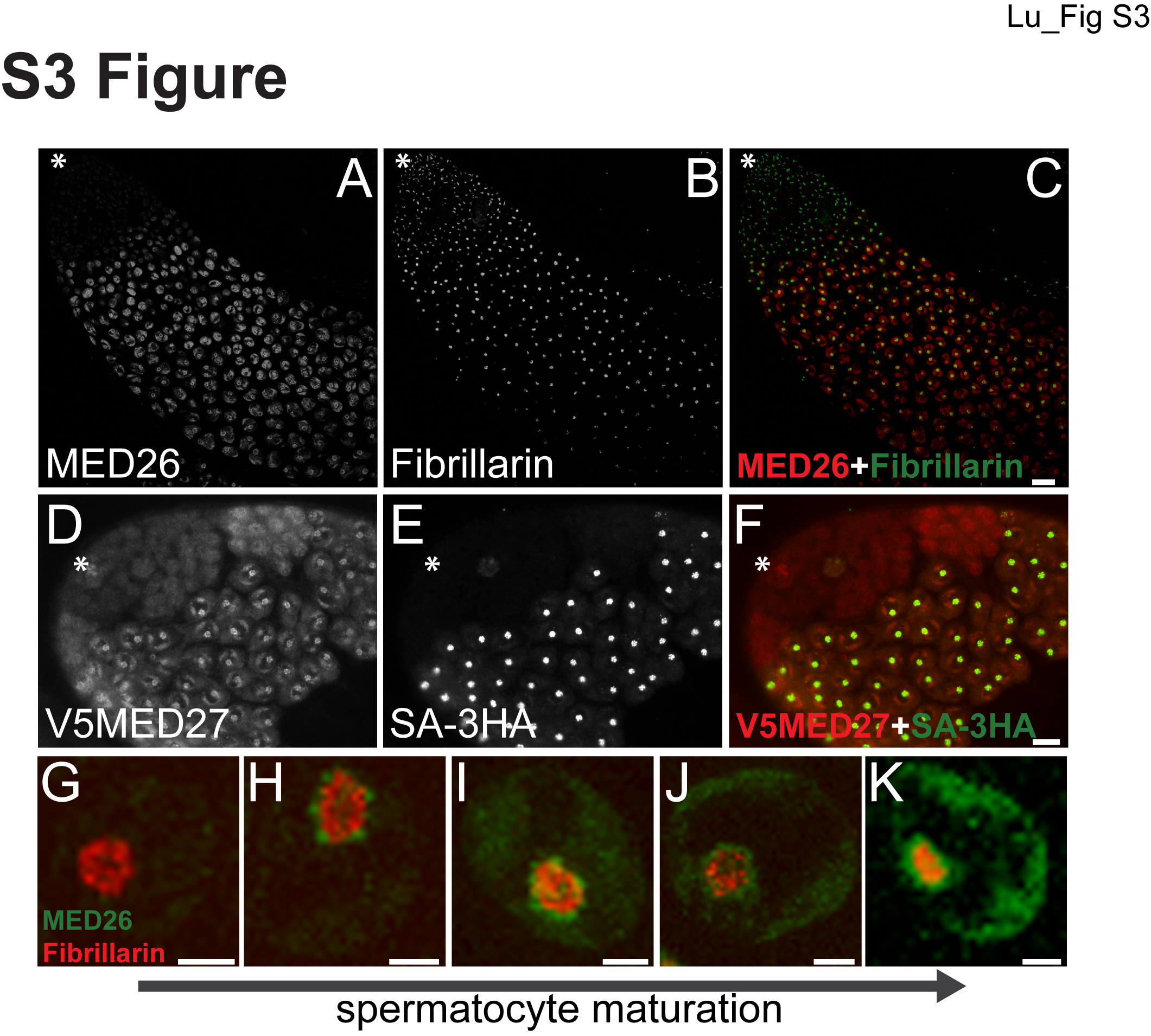

Supplement: S3 Fig — (A-C) Indirect immunofluorescence of testis showing elevated levels of MED26 accumulation in spermatocytes (A) anti-MED26, (B) anti-Fibrillarin, (C) merge, red: MED26, green: Fibrillarin. Asterisks: tip of testis. Bar: 25 μm. (D-F) V5-MED27 colocalize with SA-3HA in spermatocytes. (D) anti-V5 to detect V5-MED27, (E) anti-HA to detect SA-3HA, (F) merge, red: V5-MED27, green: SA-3HA. Asterisks: tip of testis. Bar: 10 μm. (G-K) merged images of MED26 (green) and Fibrillarin (red) showing MED26 protein enriches and gradually concentrates onto meiotic chromatin and nucleolus as spermatocyte develop from (G) young through (K) more mature stages. Bars: 4 μm. (TIF) [file pgen.1005701.s003.tif]

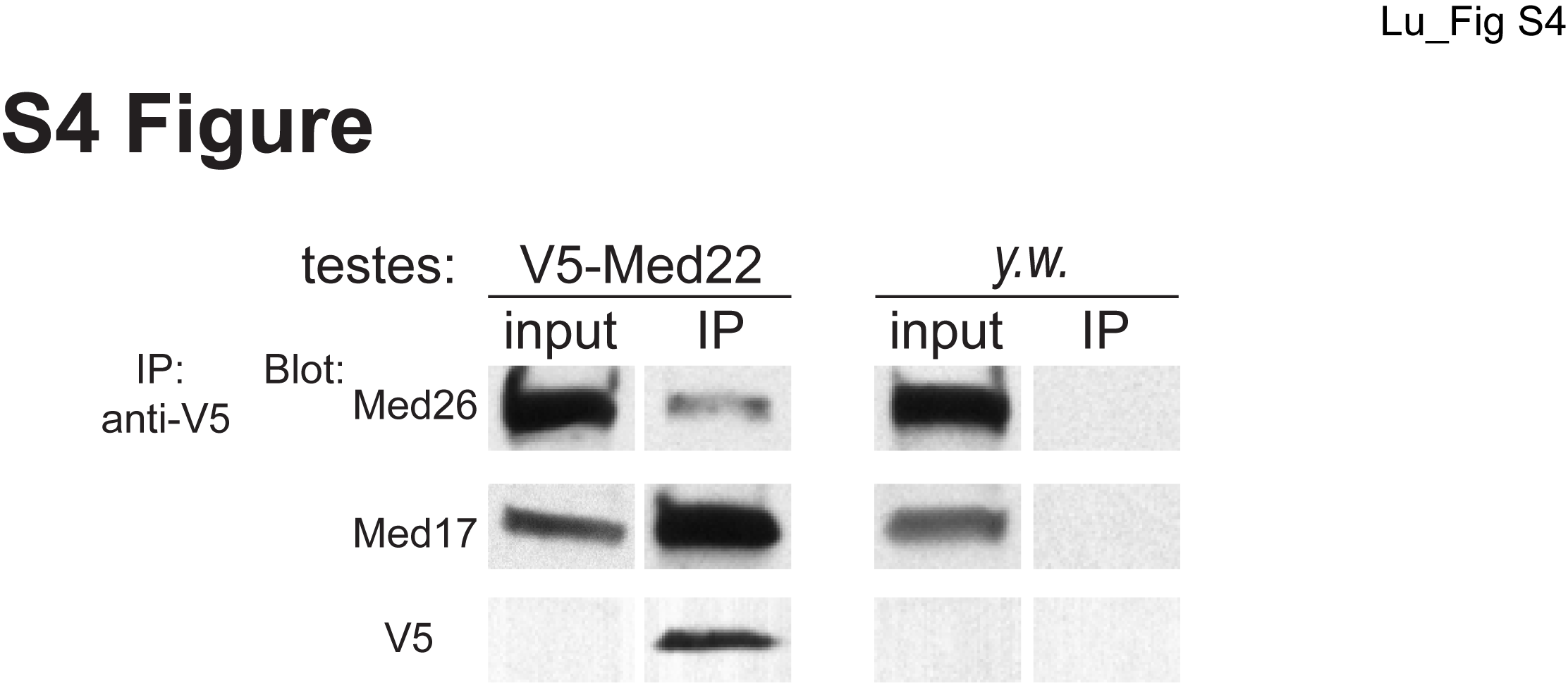

Supplement: S4 Fig — Testis extracts of V5-MED22 or y.w. were immunoprecipitated with anti-V5 and blotted with anti-MED26 or anti-MED17. 200 pairs of testis used per IP. Input is 1/10 of each pre-immunoprecipitation crude cell extract. V5-MED22 was not visible by anti-V5 blot from the V5-MED22 input (crude extract from equivalent of ~20 pairs of V5-MED22 testes) but was visible by anti-V5 blot after anti-V5 IP. (TIF) [file pgen.1005701.s004.tif]

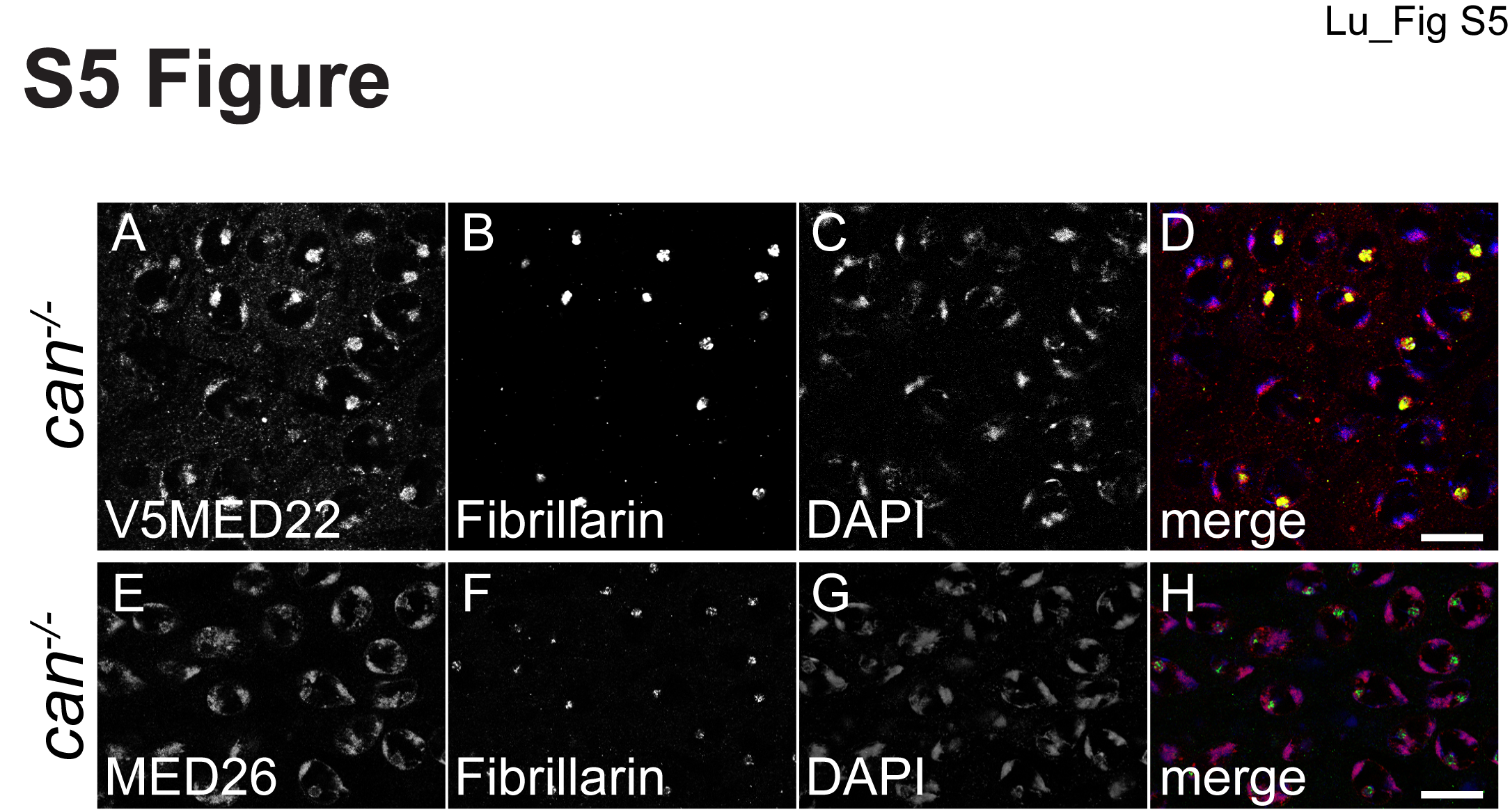

Supplement: S5 Fig — (A-H) Indirect immunofluorescence of can -/- spermatocytes stained for (A) anti-V5 to detect V5-MED22, (E) anti-MED26, (B and F) Fibrillarin, (C and G) DAPI and (D and H) merge, red: in (D) V5-MED22, in (H) anti-MED26, green: Fibrillarin, blue: DAPI. Bars: 10 μm. (TIF) [file pgen.1005701.s005.tif]

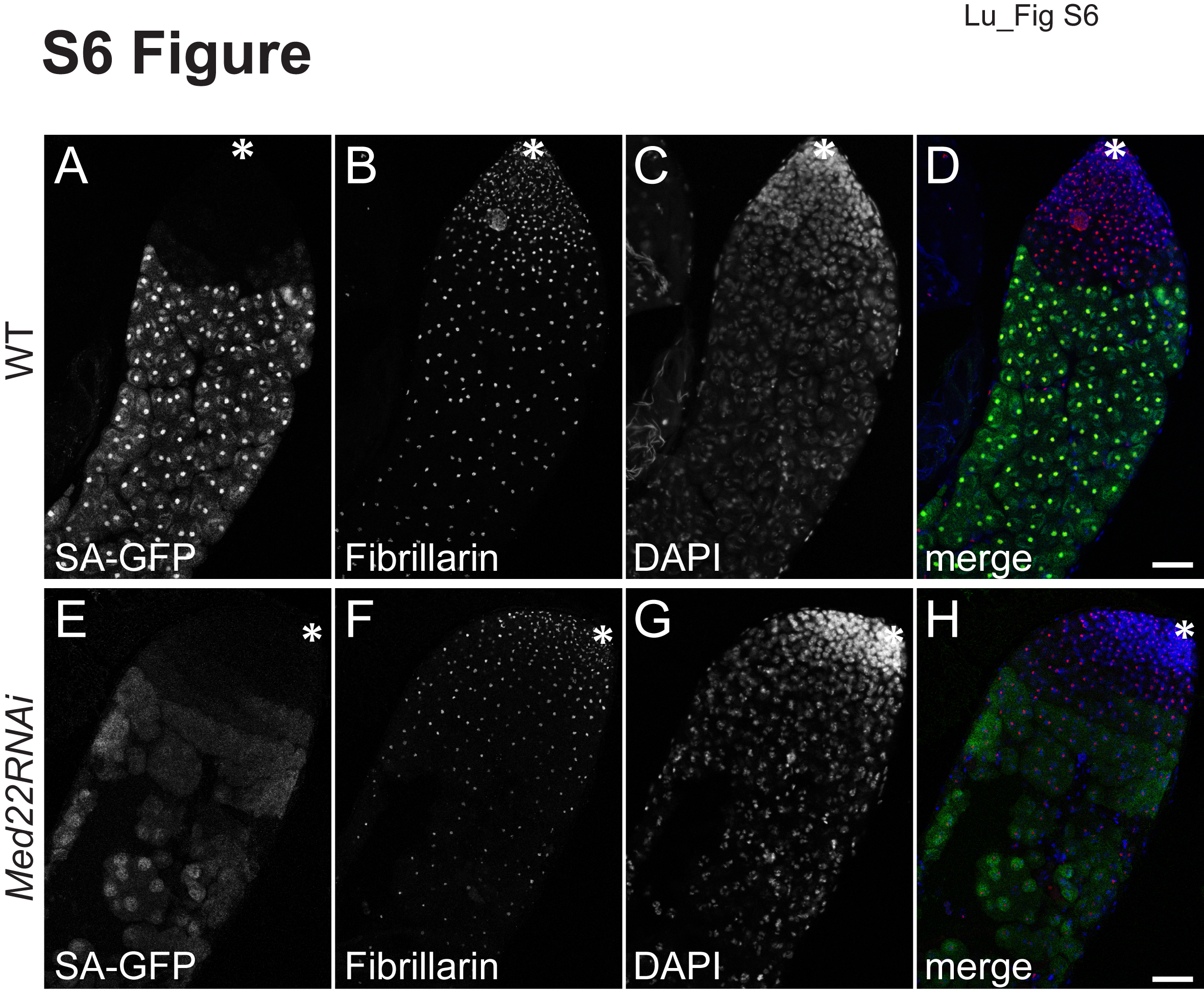

Supplement: S6 Fig — Asterisks: tip of testis. Bars: 50 μm. (TIF) [file pgen.1005701.s006.tif]
