# Supplementary material for: Recruitment of Mediator Complex by Cell Type and Stage-Specific Factors Required for Tissue-Specific TAF Dependent Gene Activation in an Adult Stem Cell Lineage
Source: PLoS Genet. 2015 Dec 1;11(12):e1005701. doi: 10.1371/journal.pgen.1005701 (PMC4666660; doi:10.1371/journal.pgen.1005701)
Supplement: S1 Table — (PDF) [file pgen.1005701.s007.pdf]

S1 Table. Summary of Mediator RNAi phenotypes

Mediator subunit	CG	VDRC RNAi #	Testis phenotype with Bam-Gal4[a]
Med22	CG3034	104581	meiotic arrest
Med17	CG7957	105264	meiotic arrest
	CG7957	44027	meiotic arrest
Med1	CG7162	108106	Incomplete arrest[b]
Med4	CG8609	14032	Incomplete arrest
Med6	CG9473	108942	Incomplete arrest
Med7	CG31390	11504	Incomplete arrest
Med8	CG13867	107783	Incomplete arrest
Med9	CG5134	41562	Incomplete arrest
Med10	CG5057	103650	Incomplete arrest
Med11	CG6884	106766	Incomplete arrest
Med14	CG12031	105163	Incomplete arrest
Med20	CG18780	52484	Incomplete arrest
Med21	CG17397	13667	Incomplete arrest
Med27	CG1245	106703	Incomplete arrest
	CG1245	13697	Incomplete arrest
Med30	CG17183	105743	Incomplete arrest
Med31	CG1057	101488	Incomplete arrest
Med12	CG8491	23142	No phenotype
	CG8491	23143	No phenotype
Med15	CG4184	21809	No phenotype
Med16	CG5465	14916	No phenotype
Med18	CG14802	106760	No phenotype
Med19	CG5546	103926	No phenotype
Med23	CG3695	105247	No phenotype
Med24	CG7999	15878	No phenotype
Med25	CG12254	108249	No phenotype
Med26	CG1793	51476	No phenotype[c]
Med28	CG5121	108282	No phenotype
Med29	CG13201	103405	No phenotype
Med31	CG1057	42444	No phenotype
	CG1057	107482	No phenotype

a. All hairpins were driven under UAS-Dicer2;;Bam-Gal4 for expression in spermatocytes. Testis phenotypes were examined by phase contrast microscopy.
b. Incomplete arrest: spermatocytes accumulate to 2/3 of testis with a few spermatid bundles near the proximal end.
c. Med26 protein was still present in Med22RNAi suggesting inefficient knock down (S3 Figure)
